# Study of Combined Ultrasound-microwave Effect on Chemical Compositions and *E. coli *Count of Rose Aromatic Water

**Published:** 2018

**Authors:** Sajad Rostami, Mehrsa Behruzian, Bahram Hosseinzadeh Samani, Zahra Lorigooini, Tahereh Hosseinabadi, Hemad Zareiforoush, Ava Behruzian

**Affiliations:** a *Department of Mechanical Engineering of Biosystem, Shahrekord University, Iran. *; b *Medical Plants Research Center, Basic Health Sciences Institute, Shahrekord University of Medical Sciences, Iran.*; c *Department of Pharmacognosy and Pharmaceutical Biotechnology, School of Pharmacy, Shahid Beheshti University of Medical Sciences, Tehran, Iran.*; d *Department of Agricultural Mechanization Engineering, University of Guilan, Rasht, Iran.*

**Keywords:** Pasteurization, Ultrasound, Microwave, Phytochemical properties, Rose water

## Abstract

Since the rose water is used in food, pharmaceutical, and cosmetic products, its microbiological control is necessary. Conventional pasteurization methods cause undesirable changes in taste, smell, medicinal properties and nutritional value with decreasing the amount of essential oil, because of high temperatures. In this study, the effects of the microwave power, temperature, ultrasound power, and ultrasonic exposure were evaluated during rose water pasteurization process on its chemical compositions and *E. coli* content. In order to determine the microbial inactivation by microwave and ultrasound, *E. coli* at a concentration of 2 × 10^6^ per mL was inoculated to rose aromatic water. The results showed that each variable on the inactivation of *E. coli* and energy consumption per microbial reduction cycle had a significant effect. The optimum values of microwave power, temperature, ultrasound power, and ultrasound exposure time were obtained 326.24 W, 43.32 °C, 100 W and 4 min, respectively. The chemical composition assessment was done by GC/MS analysis. Phenethyl alcohol is one of the main components of rose water which was completely lost in the conventional pasteurization method, while in pasteurization process by combined method, it showed an acceptable decrease as compared with raw rose water. Furthermore, the proposed method caused minimal changes in the chemical compositions of the rose water as compared to the conventional heating methods.

## Introduction

The history of medicinal plants usage and increasing global demand for use of them in the treatment of diseases due to the changing attitudes of people towards chemical drugs, has caused more attention and research of these plants in various fields, including production, processing, consumption and also phytochemical evaluation (1, 2).

One of the most widely used forms of medicinal plants is their aromatic water. The aromatic water is aqueous solution of a volatile oil or other volatile substance prepared by distillation from a plant or a mixture of different plants (3). One of the most famous aromatic water is rose water (Rose Aromatic Water), which is the main product of Rose flower with a scientific name of *Rosa damascene.* It is a traditional product that has long history in Iran and global reputation (4). The value of this product depends on the amount of microorganisms present, the essential oil content, and its naturalness. In some cases, rose water corruption is associated with turbidity. In case of aromatic water contamination with pathogens, these bacteria can be replicated in this environment. The contamination of herbal aromatic water is usually caused by raw materials, used water, packaging containers, and incomplete thermal processing. In addition to industrial applications, rose water is used to treat rheumatic and cardiovascular pains, as well as in food industries like baking pastries making (5-7). Hence, considering the use of rose water in the food, pharmaceutical, and cosmetic industries, its microbiological control is essential in order to maintain consumer health and prevent corruption (8). Therefore, pasteurization, before or after packaging, is one of the important steps in rose water production stages. Pasteurization is a process in which microbes found in foods such as bacteria, viruses, and mould are killed or their growth slowed down (9). One of the conventional methods for microbial inactivation of products is pasteurization with thermal treatment that increases the shelf life; however, using high temperature causes undesirable changes in taste, colour, odour, and medicinal properties, as well as a nutritional quality decreasing and essential oil content (10-11). Nowadays, new methods are taken into consideration to minimize the changes in the nutrient and organoleptic characteristics of products (12). The new methods can include non-thermal treatments such as ultrasound, high hydrostatic pressure, electrical field, and combined methods (13-17).

Ultrasound waves referred to as sound waves with, frequencies higher than the upper audible limit of human hearing. Usually, the highest frequency for human hearing is approximately 20 kHz (16). The mechanism of destruction of microbes is thinning of the cell membranes and production of free radicals. Researchers believe that cavitation causes mechanical effects and sonochemical reactions such as Creation of free radicals and molecular compounds, as well as oxygenated water. In general, cavitation is a phenomenon resulting in the production of a large number of micro bubbles in a liquid when there is a negative pressure. When sound waves propagate in a liquid environment, they create dense and sparse areas.

The intermolecular distance extends between liquid molecules along these waves. At very low pressure in sparse areas of liquid, intermolecular distances exceed critical molecular distance and split liquid to form empty spaces or micro-bubbles. These microbubbles oscillate with motion of the wave and become larger by connecting other micro-bubbles (19).

After several cycles, these micro-bubbles grow as the unstable size and suddenly collapse by releasing a large amount of energy and producing temperatures higher than 5000 °K. The collapsed bubbles, cause severe shear forces in the fluid which is being stirred with pressure and this consequently splits adjacent particles (20). Different studies reported the ability of ultrasound waves for inactivation of microorganism and enzymes (21). Valero *et al.* (2007) examined the effect of ultrasound and mild temperature on the active and inactive microorganisms in orange juice during the sonication process and afterwards. It was found that use of ultrasound in combination with other methods was effective to reduce the microbial activity and increased storage time (22).

One of the other new technologies which is used for food processing is microwave heating. Microwaves are a part of the electromagnetic spectrum that have a frequency between 300 MHz to 300 GHz. But the most commonly used frequencies are 915 MHz and 2450 MHz, which have wavelengths in the range of 12 to 34 cm, respectively (23). Unlike infrared waves, microwaves have not thermal energy. Microwave heating is due to food ability to absorb microwave energy and convert them into the heat. In conventional methods, the heat from an external source is transferred to the food, but the microwave heating is due to ion conduction or dipole rotation in the foods (24). Various applications of microwave have been studied extensively in food industry such as drying, melting, pasteurization, *etc.* (24, 25). 

Microbial inactivation using microwave radiation on various liquids such as apple juice, coconut water, grape juice, and milk has been also carried out. The results showed that by considering of dielectric properties, proper conditions can be achieved for applying microwave energy in the desired process (25). 

The above mentioned methods showed different effects on the microorganisms and enzymes depending on the type of products. Therefore, it seems that using combined methods can solve defect of the mentioned methods. One of the combined methods is thermo-sonication. In this method, a combination of temperature and ultrasound is applied to the foods (10).

Based on the studies and search in the scientific literature, several studies were carried out on the use of new methods to destroy microorganisms in fruits, vegetables, juices and dairy. However, there was no report about the study of the new methods effects on the properties of medicinal plant aromatic waters. Therefore, introduction and feasibility study to find an alternative method for rose water pasteurization with minimum effect on product quality is essential. In this study, it was attempted to pasteurize rose water using a combined system of microwave radiation and ultrasound device while preserving the medicinal properties and nutritional value of the product. The *E. coli *was considered to investigate the effect of ultrasound and microwave on the amount of microorganisms in rose water. *E. coli* is grown in some herbal aromatic waters, fruit juices, and food products. It is a toxic substance which can cause death in humans (26). For this reason, in the Iranian National Standard No. 3270, the destruction of *Escherichia coli* is suggested for rose water pasteurization.

## Experimental

*Pasteurization system design*

**Figure 1:**
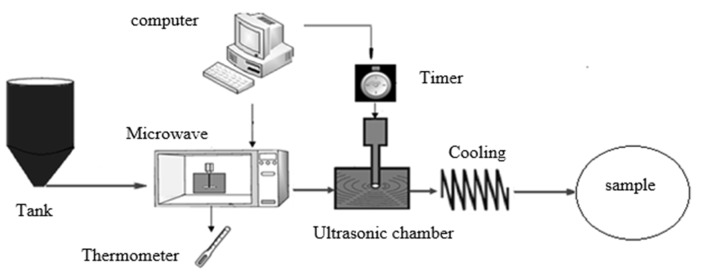
Overall scheme of combined pasteurization system

In this study, an ultrasound generator with a maximum power of 400 W and a frequency of 20 ± 0.5 kHz (Ultrasonic Technology Development Company, Iran) was used. The choice microwave oven was a domestic type (2450 MHz, 800 W, Samsung) with the volume of 2 L. The unpasteurized samples of rose water were purchased from Kaaba Company, Iran. Figure 1 shows a scheme of the pasteurization system.

The first part of the system was a primary storage tank for aromatic waters. With starting the system, rose water was poured into the microwave oven by gravity force. The microwave power was used for aromatic water preheating. By reaching the sample temperature to the set point and providing the selected power, the product was transferred from microwave to the ultrasonic reactor to perform the ultrasound treatments. In the microwave compartment, two holes were made to pass the connecting tubes. Since the holes in the microwave chamber could cause emission of waves into the environment, the aluminium foils were used in the holes to isolate the chamber. The safety of the chamber was then investigated using a leak detector device (27).

*Microbial Test*

Once the lyophilized standard strain of *E. coli* vial was opened, the strain was cultivated on an agar nutrient culture (Micromedia, Hungary). Therefore, a loop of grown microbial strain on agar was inoculated with a 25 mL nutrient liquid culture (NLC) under sterile conditions in order to prepare the microbial suspension. The final concentration of *E. coli* in the inoculum was determined by plating serial dilutions on NLC and incubating at 37 °C for 24 h. The liquid culture containing the grown cells was centrifuged for 5 min at speed of 8000 rpm. Afterwards, the resulting cellular mass was suspended in the sterile aromatic water sample. A total of 3 mL of this suspension was inoculated into 300 mL of aromatic water sample to a final concentration of 2 × 10^6^ per mL. For adaptation purpose, it was held for 15−30 min before the deactivation (11, 22).

*Qualitative testing*

*Isolation of essential oil from rose aromatic water*

Aromatic water (100 mL), come out from each treatment, was shaken and then decanted three times with diethyl ether (50 mL) in a separator funnel. The organic phases were separated, and then the diethyl ether was evaporated. To absorb water along with the essential oil, anhydrous sodium sulphate (Na_2_SO_4_) was used. Remain solvent was evaporated by nitrogen gas to get concentrated oily fragrant volatile fraction. It was stored at 4 °C and dark place for next analyses process (28).

*Chemical analysis*

GC analysis was done using a Thermoquest gas chromatograph with a flame ionization detector (FID). The analysis was carried out using a fused silica capillary DB-5 column (30 m × 0.25 mm; film thickness 0.25 µm). The injector and detector temperatures were kept at 250 °C. Helium was used as carrier gas with flow rate of 1.1 mL/min; oven temperature program was 60–250 °C at the rate of 5 °C/min, and finally held isothermally for 10 min; the split ratio was 1:100.

GC/MS analysis was performed using a Thermoquest-Finnigan gas chromatograph equipped with the same column, coupled with a TRACE mass ion trap detector (Manchester, UK). Helium was used as carrier gas with an ionization voltage of 70 eV. Ion source and interface temperatures were 200 °C and 250 °C, respectively. The mass range was from 40 to 460 m/z. Oven temperature program was the same as the mentioned above for the GC. After injection, the essential oil ingredients were isolated by the gas chromatography and then the compounds were identified by mass spectrometer. Linear hydrocarbon series 8-24 were also injected into the gas chromatography at the same conditions. Essential oil compounds were identified based on the comparisons of Retention Time (RT), Kovat›s Index (KI), and mass spectra of each compound with libraries of Wiley and Adams 2007 (29).

*Analysis, Modeling and Optimization*

The optimum conditions to minimize the microorganisms and energy consumption during the pasteurization process were determined using Response Surface Methodology (RSM) (30). Box-Behnken with five center points was used in this experiment. To analyze the response surface method, the Design Expert 7 software was utilized. The variation range of independent variables were selected (Table 1).

The dependent variables were defined as residual *E. coli *(bacteria that can cause death of any person), energy consumption, and energy consumed per cycle.

In order to obtain the optimum value, Equation 1 was used:

Y_i_ = β_0_ + ∑ Β_i_ X_i_ + ∑β_ij_X_i_X_j_ + ∑β_jj_X^2^_i_ + ε                                       (1)

Where βo, βi, βij and βjj are constants, xi and xj are independent variables in the process, and ε is a random error.

*Data evaluation and comparison with conventional method*

The prepared samples were placed in a water bath (85 °C) for 1 min to simulate the common method of pasteurization. Then, the chemical composition of raw rose water in the conventional and proposed pasteurization methods was compared.

## Results and Discussion

*E. coli Survival Counts and energy per cycle*

The effects of variables, including ultrasound power, microwave power, sample temperature, and ultrasonic exposure time on the *E. coli* count in the rose water were investigated. Finally, the results were modelled and optimized. Analysis of variance (ANOVA) for the quadratic model showed that this model had a significant reduction on the *E. coli* count in rose water samples. The non-significance of lack of fit shows the suitable accuracy of the resulted model (Table 2).

The results showed that all coefficients of variables in the model were significant at 10% level except for interactions of ultrasound power × temperature, microwave power × ultrasound power, microwave power × exposing time, squared ultrasound power, and square exposing time.

According to Table 2, some coefficients of model are not significant for microbial reduction. Therefore, in order for simplification, they were removed from the fitted model. The adjusted coefficient of determination, coefficient of variation (C.V) and standard error of the model were 0.9934, 83%, and 0.031, respectively. The final equation was obtained as follows (2):

$\log\left(\frac{N}{N0}\right)=$-3.77 - 0.098 × MP - 0.28 × UP - 0.45 × Temp - 0.13 × t - 0.061 × MP × Temp + 0.044 × Temp × UP -0.056 × Temp × t - 0.046 MP^2 ^+ 0.17 × Temp^ 2^                                       (2)

Where, N_0_ is the initial number of *E. coli* (CFU/mL), N is the secondary number of *Escherichia coli *(CFU/mL), MP is Microwave Power (W), Temp is temperature of rose water (°C), UP is ultrasound power (W), and t is ultrasound exposure time (min).

**Figure 2:**
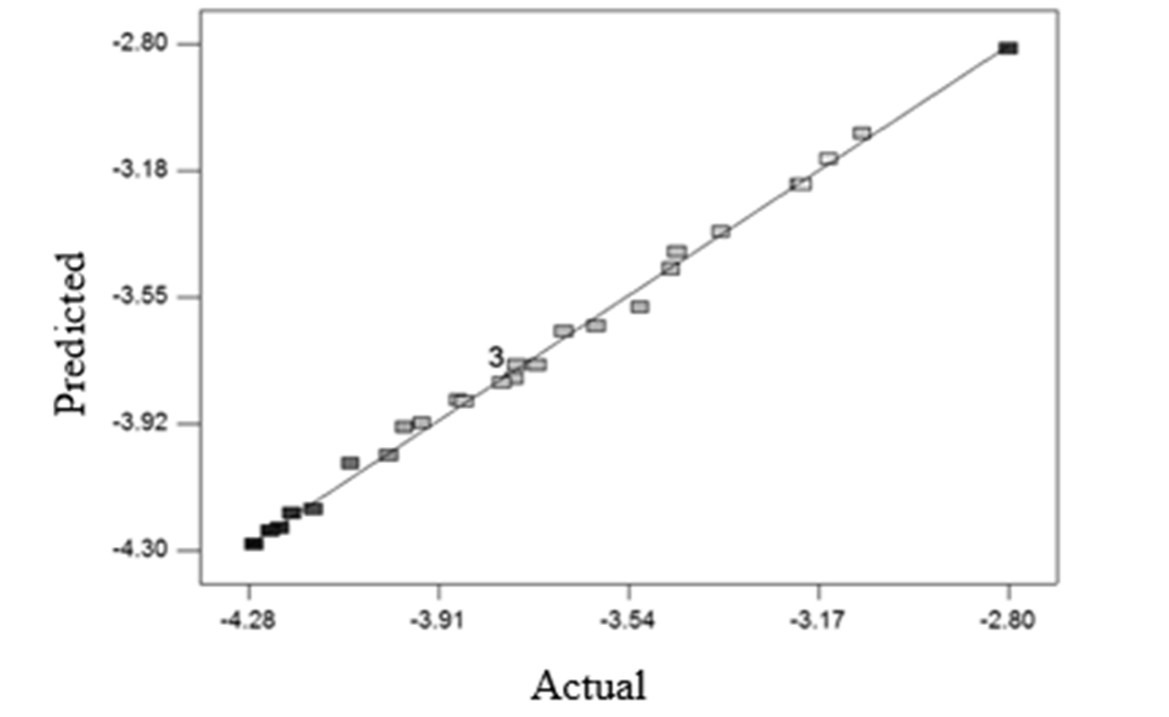
Fitting experimental data and model data for reduction of *E. coli*

Figure 2 shows the proper fitting of the experimental data and data obtaind from the model for microbial count reduction.

Variance analysis of quadratic model showed the significance of the full quadratic model in data obtained for the ratio of energy changes per a cycle of *E. coli* reduction in the rose water sample. The non-significance of lack of fit shows the suitable accuracy of the resulted model (Table 3).

According to Table 3, all coefficients of model except for the interaction of microwave power × ultrasound power were significant at 10% level. Therefore, in order to simplify, the interaction of microwave power × ultrasound power was removed from the fitted model. The adjusted values for the coefficient of determination, the coefficient of variation (C.V) and standard error of the new model were respectively equal to 0.9998, 0.32, and 0.65. The final equation was obtained as follows (4):

energy/cycle=481.37 - 10.99 × MP + 216.94 × UP - 55.71 × Temp + 109.90 × t - 5.71 × MP × Temp - 16.52 × MP × t - 30.65 × Temp × UP + 46.67 × UP × time - 19.66 × Temp × t - 8.40 MP^2 ^+ 48.96 × UP^2 ^+ 31.04 × Temp^2 ^- 7.56t^2^                                       (3)

**Figure 3:**
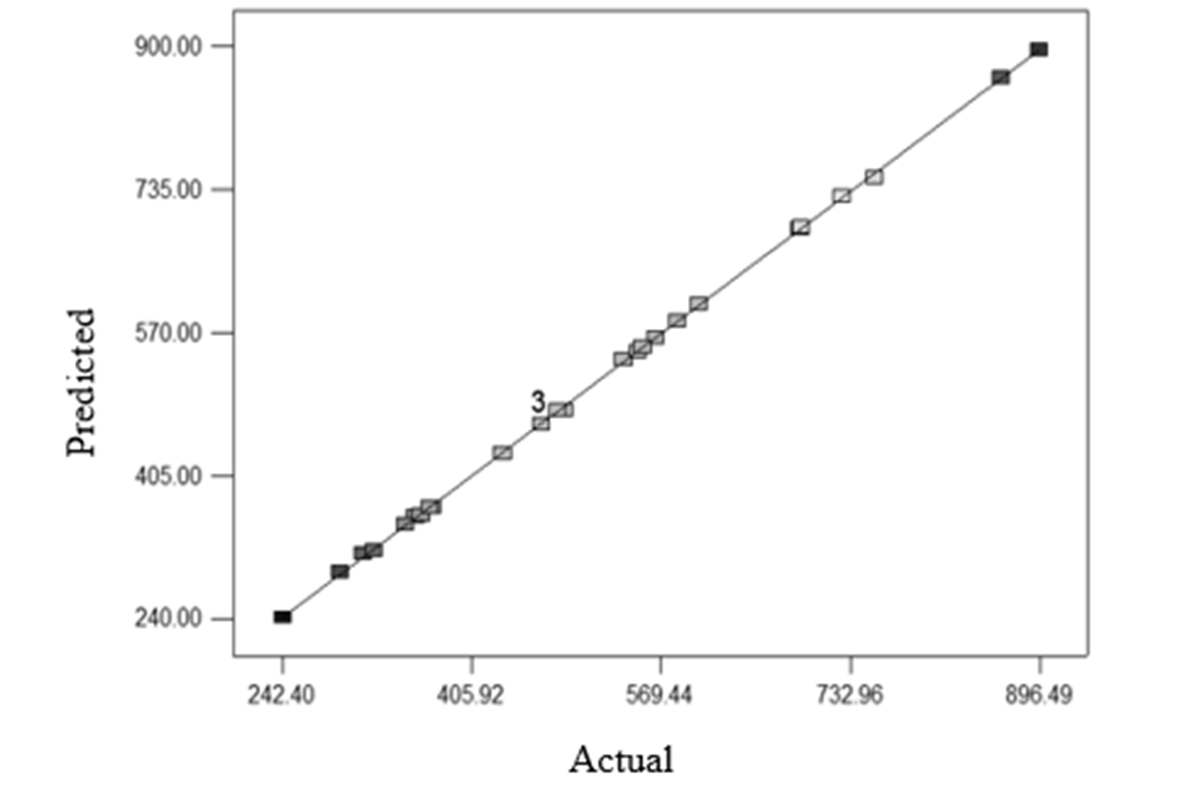
Fitting experimental data and model data for energy changes in one cycle of *E. coli *reduction

Figure 3 shows the proper fitting of the experimental data and data obtained from the model for the ratio of energy changes in a cycle of *E. coli* reduction (energy/cycle).

**Table 1:** Selected levels of independent variables in response surface methodology

**Independent variable**	**Range of level**		
	**-1**	**0**	**1**
Microwave power (W)	300	450	600
Temperature (°C)	30	45	60
Ultrasound power (W)	100	150	200
Ultrasonic exposure time (min)	2	4	6

**Table 2:** ANOVA for the coefficients of quadratic model in the method of response surface curves for logarithmic reduction of *E. coli*(CFU/mL) in rosewater

**Source**	**Df**	**Sum of Squares**	**Mean Square**
Model	14	4.02	0.29**
Microwave power	1	0.12	0.12**
Temperature	1	2.44	2.44**
Ultrasonic power	1	0.96	0.96**
Time	1	0.21	0.21**
Microwave power × Temperature	1	0.015	0.015**
Microwave power × Ultrasonic power	1	1.397E-003	1.397E-003**
Microwave power × Time	1	1.489E-004	1.489E-004**
Temperature × Ultrasonic power	1	7.720E-003	7.720E-003**
Temperature × Time	1	0.013	0.013**
Ultrasonic power × Time	1	1.341E-004	1.341E-004**
Microwave power × Microwave power	1	0.017	0.017 ns
Temperature × Temperature	1	0.19	0.19**
Ultrasonic power × Ultrasonic power	1	1.014E-003	1.014E-003**
Time × Time	1	1.504E-003	1.504E-003 ns
Residual	14	0.014	1.027E-003
Lack of Fit	10	0.013	1.309E-003 ns
Pure Error	4	1.280E-003	3.201E-004**
Cor Total	28	4.03	

** Showed a significant effect at 10% level.

**Table 3:** ANOVA of coefficients of quadratic model in the response surface method for the ratio of energy changes in a cycle of *E. coli* reduction (energy/cycle) in rosewater

**Source**	**Df**	**Sum of Squares**	**Mean Square**
Model	14	7.875E+005	60575.63**
Microwave power	1	1448.68	1448.68**
Temperature	1	37247.04	37247.04**
Ultrasonic power	1	5.647E+005	5.647E+005**
Time	1	1.449E+005	1.449E+005**
Microwave power × Temperature	1	130.47	130.47**
Microwave power × Ultrasonic power	1	14.15	14.15 ns
Microwave power × Time	1	1092.24	1092.24**
Temperature × Ultrasonic power	1	3758.85	3758.85**
Temperature × Time	1	1545.75	1545.75**
Ultrasonic power × Time	1	8636.26	8636.26**
Microwave power × Microwave power	1	457.28	457.28**
Temperature × Temperature	1	6250.87	6250.87**
Ultrasonic power × Ultrasonic power	1	15549.99	15549.99**
Time × Time	1	371.19	371.19**
Residual	14	89.82	5.99
Lack of Fit	10	68.47	6.22 ns
Pure Error	4	21.35	5.34**
Cor Total	28	7.876E+005	

** Showed a significant effect at 10% level.

**Table 4:** Boundary conditions to optimize the reducing of microbial load

**Name**	**Goal**	**Lower Limit**	**Upper Limit**	**Lower Weight**	**Upper Weigh**	**Importance**
Microwave power (W)	In range	300	600	1	1	3
Temperature (°C)	In range	30	60	1	1	3
Ultrasonic power (W)	In range	100	200		1	3
Time (min)	In range	2	6	1	1	3
*E. coli *count	Target	-6	-2.80434	1	1	4
energy/cycle	Minimize	242.659	896.487	1	1	3

**Table 5:** Effect of different pasteurization methods on chemical constituents of Rose aromatic water

**No**	**RI** a	**Compound**	**Rose water oil** b **with No treatment**	**Rose water oil with Conventional Method**	**Rose water oil with Combination method**
1	998	n-Decane	Tr^c^	Tr	Tr
2	1025	p-Cymene	Tr	Tr	Tr
3	1029	Limonene	Tr	Tr	Tr
4	1031	1,8-Cineole	Tr	Tr	Tr
5	1036	Z-β-Ocimene	Tr	Tr	Tr
6	1058	Terpinene<gamma->	Tr	Tr	Tr
7	1102	Linalool	1.17	-	0.69
8	1122	Phenethyl alcohol	48.28	-	30.71
9	1160	Terpineol<cis-dehydro-beta->	0.82	-	0.46
10	1188	Cryptone	1.17	-	1.28
11	1194	Terpinene<alpha>	2.45	-	-
12	1231	β-Citronellol	0.97	-	-
13	1233	7-Octene-2,6-diol, 2,6-dimethyl	0.24	-	-
14	1247	Carvone	1.82	-	4.47
15	1300	Menthone<iso->	Tr	Tr	Tr
16	1302	cis-Dihydrocarvone	Tr	Tr	Tr
17	1312	Methyl chavicol	Tr	Tr	Tr
18	1362	trans-Dihydrocarvone	1.25	-	-
19	1369	Pulegone	2.30	-	2.09
20	1398	Carvone	Tr	Tr	1.57
21	1408	2,4-Di-tert-butylphenol	0.50	-	0.39
22	1520	Myristicin	2.05	0.82	0.85
23	1704	Unknown	6.14	9.13	8.58
24	1708	n-Heptadecane	15.58	42.63	22.96
25	1712	Unlnown	1.72	3.61	2.58
26	1719	(2*E*,6*Z*)-Farnesol	2.78	9.65	5.26
27	1729	Neocnidilide	3.31	7.11	4.71
28	1749	Tridecane, 2-methyl- (CAS)	3.62	12.07	5.85
29	1923	Unknown	3.14	10.64	5.32
30	1929	Unknown	0.60	-	-

a RI: Retention Index;

b Relative percentage (%) obtained on DB-5 column cappilary column;

c Tr (trace) =< 0.1%.

**Figure 4:**
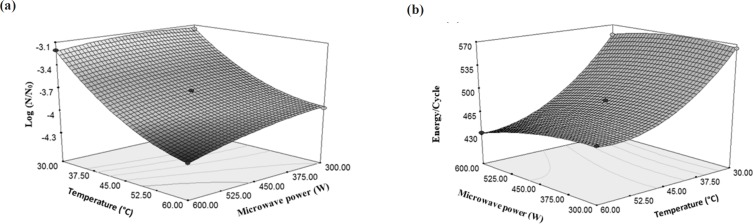
Changes of in (a) logarithmic reduction of *E. coli *and (b) energy/cycleunder the influence of microwave power and temperature

**Figure 5:**
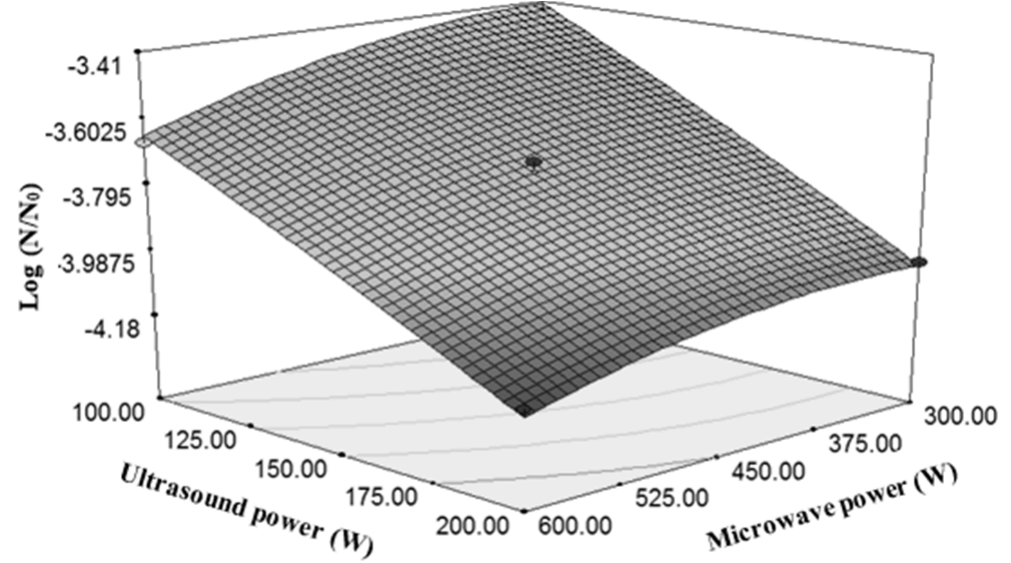
Changes in logarithmic reduction of *E. coli *influenced under the influence of microwave power and ultrasound power

**Figure 6:**
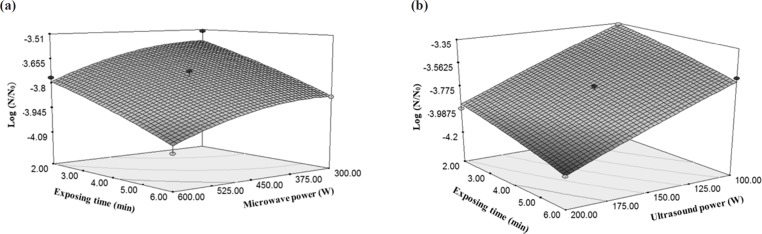
Changes of in logarithmic reduction of *E. coli *under the influence of (a) microwave power and exposing time, (b) ultrasound power and exposing time

**Figure 7:**
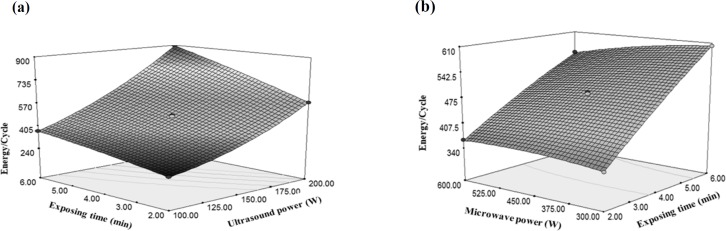
Changes of in energy/cycle under the influence of (a) ultrasound power and exposing time, (b) microwave power and exposing time

**Figure 8:**
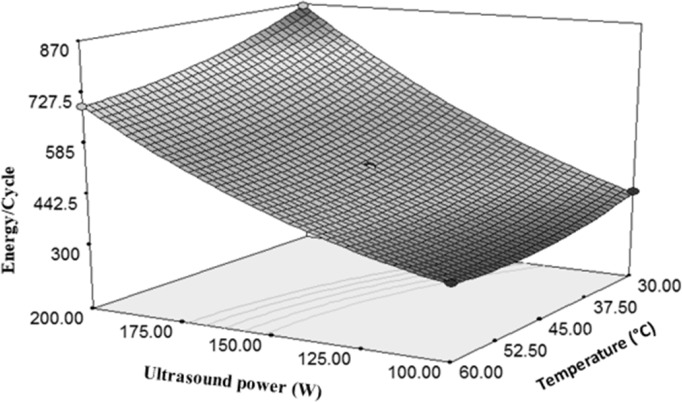
Changes of in energy/cycle under the influence of ultrasound power and temperature

**Figure 9:**
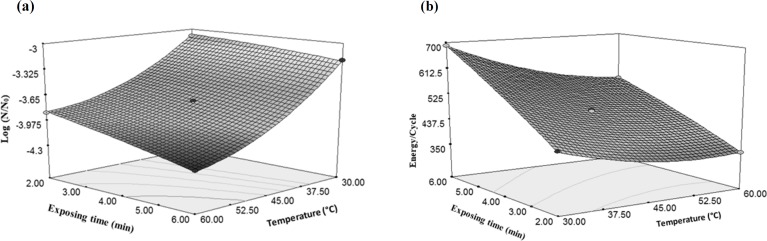
Changes in (a) logarithmic reduction of *E. coli *and (b) energy/under the influence of exposing time and temperature

**Figure 10:**
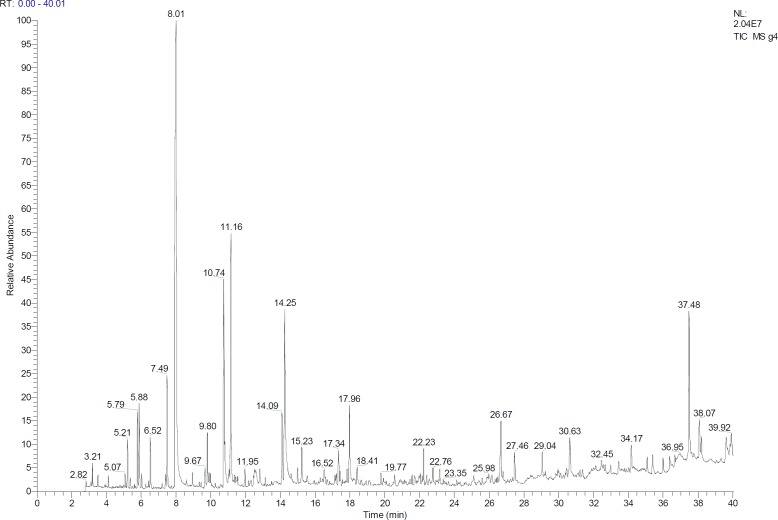
Chromatogram GC-MS of no treatment sample

According to the results, the importance of final temperature of rose water to reduce *E. coli* was more than the microwave power (producing factor of temperature); because increasing the temperature has led to an increase in the gradient of microbial decreasing but, increase in the microwave power has milder gradient than temperature (Figure 4a). Increasing the power of the microwave from 300 W to 600 W caused 5.59% decrease in the total microbial count, but increasing temperature from 30 °C to 60 °C reduced the microbial count by 28.7%. However, increasing the temperature to 60 °C by the microwave power (because of the higher speed of reaching to the mentioned temperatures and lacking sufficient time for microorganisms to be adapted with the new conditions), increased the reduction of *E. coli *(31). These results can be found from Equation 2 that multiplying the microwave power by the temperature has a negative coefficient. This means that with increase in the amount of two mentioned independent variables, the result of Equation 2 would be a greater negative number which indicates a more reduction in the amount of *E. coli*. Destruction of microbes or enzymes by microwaves in lethal temperature is due to the selected heating property and interruption of cell membranes. Microorganisms were selectively heated due to the dielectric property, which causes them to become warmer in comparison with the temperature of the surrounding liquid, and consequently, collapse of the cellular structure (32).

The effect of temperature on energy changes per a cycle of *E. coli *inactivation was higher than the effect of temperature created by microwave power. Increasing the microwave power from 300 W to 600 W did not have a significant effect on the energy changes per a cycle while increasing the temperature from 30 °C to 60 °C caused 24% reduction in consumed energy per each cycle (Figure 4b).

The effect of ultrasound power was higher than microwave power in *E. coli* reduction (Figure 5). Twice increasing of ultrasound power from 100 W to 200 W caused 29% decrease in the total microbial count, but twice increasing of microwave power from 300 to 600 reduced the *E. coli *count by 5.59%. By increasing the ultrasound power, its lethal effects increase. The reason for this decline of *E. coli* count can be justified by the increased range of horn motion in the fluid. Increasing the motion range causes to increase the number of bubbles formed in the fluid, and thereby, an increase in the cavitation is expected (33). Several researches were carried out on the effects of ultrasound power in the reduction of microbial count. In a study on the orange juice, by applying ultrasound treatment (20 kHz, 500 W ultrasonic treatments in the ranges of 50, 60 and 75%), logarithmic cycles of mesospheric aerobic bacteria, mould and yeast were reduced by 1.38 and 0.56, respectively. Furthermore, the treated sample (75% of sound range for 8 min) showed more shelf life than the control by 4 days (34).

Results of similar study for the interaction of ultrasound and microwave for deactivation of *E. coli* in sour cherry juice showed greater effect of ultrasound power. Microwaves destroy microorganisms through thinning the cell wall, temperature concentration, and producing and spreading of free radicals into the fluid (30).

As shown in Figures 6a and 6b, increasing the ultrasound power and exposing time caused anincrease in the reducing gradient of *E. coli* as compared to the microwave power. Thus, with the increase in the ultrasound treatment time, microorganism counting decreases in the rose water. Because the increased exposing time increases the number of periods of sound streams in the reactor which results in more influences of the ultrasound waves on the *E. coli *(31). A research on the lemon water with 25 kHz sonication (70% power) for 30 and 60 min at a constant temperature of 20 ± 1 °C showed total count of microorganisms and yeasts were reduced by 1.9 and 0.5 log cycle, respectively (35).

According to Table 2, due to non-significance of squared ultrasonic power and significance of squared microwave power, it can be concluded that reducing the total number of microorganisms with respect to the changes in the ultrasonic power was linear, while the variations versus the microwave power were quadratic.

Considering the higher gradient of the ultrasonic power as compared with time changes and given the positivity of both variables, it can be deduced that the changes of ultrasonic power had greater effects on the energy changes per cycle (Figure 7a). Moreover, due to negativity and low values of microwave changes gradient versus the variations of sonication time, it can be concluded that the effect of microwave power on the energy changes per each cycle was lower than the sonication time (Figure 7b).

Both of the sample temperature and time of sonication were independent and important variables which had significant effects on the *E. coli* reduction*.* Due to more gradient of the ultrasonic power than the temperature, it can be said that the ultrasonic power can more affect the amount of energy used to deactivate microorganisms per each cycle as compared with temperature (Figure 8). Figure 8 indicates a direct relationship between the ultrasonic power and the rate of energy changes in a cycle of inactivation. It is observed that increasing the ultrasonic power from 100 W to 200 W increases the consumed energy to 58%. However, twice increasing of temperature caused 24% decrease in the energy consumption during a cycle of inactivation. In other words, it can be said that the changes in the temperature and the amount of energy consumed per a cycle were inversely related to each other. According to (Equation 4), due to the positivity of the ultrasonic power coefficient and negativity of the temperature coefficient, and also because of the higher value of the ultrasonic power coefficient, it can be concluded that the ultrasonic power had a direct effect on the energy consumption per a cycle.

As shown in Figure 9a, increasing temperature from 30 °C to 60 °C decreased E. coli as much as 28.7%, while three-folds increasing of time from 2 min to 6 min only caused a 7.3% decrease in E. coli count. Therefore, the effect of temperature on the inactivation of microorganisms can be more than time. As temperature rises, the effect of ultrasound and cavitation intensity decreases, but in general, the effect of temperature and ultrasound on E. coli reduction is more than the time when each process has been used individually. These results can be observed in another study that evaluated the effect of ultrasound and microwave on the* E. coli* count during pasteurization of sour cherry juice. The microwave (352.21 W and 49.94 °C) and ultrasound (475.13 W for 6 min) methods were used. The results showed that temperature was more effective factor to decrease the total microbial count (30).

According to Figure 9b, ultrasonic exposure time has more effect on the consumed energy changes per one cycle as compared with the temperature changes. Increasing sonication time from 2 to 6 min resulted in 60% increase in the energy consumption. According to Equation 4 it can be concluded that due to positivity of time coefficient, there is a direct relationship between the time and the amount of consumed energy per a cycle.

*Optimization*

At the end, the aimed process was optimized. The objective function was Equation 3 and the relationship between the amounts of consumed energy. The aim of this optimization was to achieve a condition for the independent variables (microwave power, sample temperature, and ultrasonic exposure time) in which with the lowest energy consumption, the amount of *E. coli* in the sample would be equal to zero. Boundary conditions in optimization process were specified so that values of independent variables were placed in the test range. All of the independent and dependent variables were assigned with identical weights and in the desirable end, minimizing the objective function was selected (Table 4). The optimum values for the microwave power, the sample temperature, ultrasound power, and ultrasound time were obtained as 326.4 W, 43.32 °C, 112.3 W, and 4.36 min, respectively. For these mentioned values, the amount of residual *E. coli* was equal to 0. Finally, to verify the optimal point, the independent variables were rounded to the nearest integer to have practical applicability. Accordingly, the microwave power, sample temperature, ultrasound power, and ultrasonic exposure time were equal to 300 W, 45 °C, 100 W, and 4 min, respectively. *E. coli* was determined in the laboratory and its value was equal to zero. This indicates the high accuracy of the analytical and optimization method.

*Chemical compositions analyses*

The data from GC/MS were used to identify the components of the samples. They are listed in the order of their elution from a DB-5 column (Table 5). Finally, the analysis of the GC (Figure 10) and comparison between the conventional thermal pasteurization method and the proposed method in this study showed that the composition of pasteurized product in the combination method was closer to the raw rose water. In total, 18-26 constituents were detected and identified in the samples. Identification was determined for 72.28–88.31% of the essential oil components. Other research results revealed that phenethyl alcohol, geraniol, and b-citronellol were the main constituents of the most samples. This study like other researches was confirmed that Phenethyl alcohol is the main component of natural rose and rose water and because of its high polarity and water solubility, it remained dissolved in the distillate water (4, 28 and 36). Phenethyl alcohol that is one of the main compounds of rose water, was completely lost in the conventional pasteurization method, while in the combined method showed an acceptable decrease as compared with raw rose water. Other compounds, such as Linalool and Cryptone were completely lost during the conventional thermal method, while they were observed in the product of the proposed method. It should be noted that some compounds such as Terpinene<alpha>, β-Citronellol, 7- Octene-2,6-diol, 2,6-dimethyl (which have small amounts in the raw rose water) were not detected in both of the conventional and combined methods. In the other research, various methods were applied to ensure the compositional quality and microbial safety of rose oil during shelf life. The methods used in the study were conventional pasteurization and ultra violet treatment, Phenoxyethanol and sodium benzoate application. Although there were minor compositional differences among methods, it was shown that all methods provided satisfactory prevention of microbial spoilage (37). Conventional pasteurization method caused to lose the chemical compounds of raw rose water and this would necessitate using non-thermal alternative methods to pasteurize rose water.

## Conclusion

Microwave power, rose water temperature, ultrasound power, and ultrasonic exposure time were effective on *E. coli* count and energy/cycle. The final temperature of the rose water in reducing *E. coli* count was more important than the microwave power that causes the temperature. The effect of microwave power on EPC was more than temperature. The ultrasound power was more effective in reducing *E. coli* in rose water as compared to the microwave power. Both sample temperature and ultrasonic duration were important independent variables and effective factors on *E. coli* reduction. The main compounds of rose water pasteurized by the combined method had acceptable decrease as compared to raw rose water. Some compounds like Lianalool and cryptone were observed in combined method pasteurized sample while were completely lost in the conventional pasteurization method. Conventional pasteurization method caused to lose the chemical compounds of raw rose water and this would necessitate using non-thermal alternative methods to pasteurize rose water.
